# Artificial intelligence reveals dysregulation of osteosarcoma and cuproptosis-related biomarkers, PDHA1, CDKN2A and neutrophils

**DOI:** 10.1038/s41598-023-32195-2

**Published:** 2023-03-26

**Authors:** Jie Jiang, Xinli Zhan, Jianxun Wei, Qie Fan, Haowen Li, Hao Li, Shuzhen Li, Yong Zhao, Guodong Yin, Lin Tang, Yongxiang Wu, Mindong Lan, Yijue Qin, Quan Guo, Weicheng Xu, Ling Lu, Yanwei Yang, Yitian Zhang, Haishun Qu

**Affiliations:** 1 Orthopedics, Guangxi Academy of Medical Sciences, Guangxi Zhuang Autonomous Region People’s Hospital, Nanning, 530016 People’s Republic of China; 2 Spinal Orthopedic Surgery, The First Affiliated Hospital of Guangxi Medical University, Nanning, 530021 People’s Republic of China; 3 Department of Traditional Chinese Medicine, Guangxi Academy of Medical Sciences, Guangxi Zhuang Autonomous Region People’s Hospital, Nanning, 530016 People’s Republic of China

**Keywords:** Bone cancer, Cancer genomics, Cancer microenvironment, Cancer models, Diagnostic markers

## Abstract

At present, the impact of cuproptosis-related genes in the study of osteosarcoma is largely unknown. Genome-wide data of osteosarcoma and controls were downloaded from 3 different databases, and specific diagnostic models associated with cuproptosis in osteosarcoma were constructed by support vector machines with artificial intelligence, random forest trees and LASSO regression. Differential analysis of immune cell infiltration was examined using routine blood data from 25,665 cases. Differential expression was examined using immunohistochemistry and PCR. PDHA1 and CDKN2A were obtained as specific cuproptosis-related biomarkers for osteosarcoma after artificial intelligence analysis. PDHA1, CDKN2A and neutrophils were differentially expressed in OS and control groups. PDHA1 and CDKN2A are significantly dysregulated in OS and are able to serve as biomarkers of OS.

## Introduction

Osteosarcoma (OS) is the number one primary malignant bone tumor among orthopedic tumors, and those who are susceptible to this disease are children and adolescents, whose 5-year survival rate is less than 20% if it metastasizes^1,2^. It has been shown that immune dysregulation in the body is associated with the development of osteosarcoma, and improving the survival of patients with osteosarcoma has long been considered challenging, although treatment of this disease has also improved in recent years, and immune checkpoint inhibition may enhance the therapeutic effect on OS by improving immunosuppression as well as the tumor microenvironment^3^. It is increasingly recognized that osteosarcoma cells, immune cells, osteoblasts and osteoclasts coexist and interact with each other in the microenvironment of osteosarcoma^4^. Because of the poor prognosis of OS, there is an urgent need to find more accurate biomarkers to guide early diagnosis.

Artificial intelligence (AI) is an emerging technology, and as technology advances, more and more methods of AI are being used in the medical field^5^. Some studies have shown that AI tools can increase the detection of precancerous polyps during colonoscopy, a technological advancement that may help prevent colon cancer in the long term^6^. The study by Mirelman et al. found that the machine learning approach had higher discriminatory value in motor disease segments by applying machine learning features and classification algorithms to differentiate between 332 Parkinson's disease patients (Hoehn and Yahr scale I–III) and 100 healthy controls^5^. However, the research and application of AI, an advanced technology in OS, is still inadequate, which requires researchers to apply AI technology to the early diagnosis of OS.

Copper is an essential factor in all organisms, and once copper concentrations exceed the threshold maintained by homeostatic mechanisms, then copper in the body becomes toxic; in human cells, copper-dependent death occurs through direct binding of copper to lipid acylated components, which leads to aggregation of lipid acylated proteins and loss of iron-sulfur cluster proteins, ultimately leading to cell death^7^. The balance of copper as an important cofactor is crucial, as dysregulation of intracellular copper bioavailability will induce cytotoxicity and oxidative stress; in both prokaryotes and eukaryotes, copper homeostasis is finely regulated with the main aim of preventing excessive accumulation of copper ions in the cell and thus threatening cell survival^8^. It is now clear that the genes CDKN2A, FDX1, DLD, DLAT, LIAS, GLS, LIPT1, MTF1, PDHA1 and PDHB are closely associated with cuproptosis as a form of mortality^7,9^. There is still a gap in the research of this novel form of death in OS.

The main objective of this study is to explore OS and cuproptosis-related and immune-related diagnostic biomarkers through an artificial intelligence approach in order to more accurately guide early clinical diagnosis and immunotherapy of this disease.

## Materials and methods

### Data download and preliminary processing

In this study, genomic expression data of osteosarcoma for the training set were downloaded from the UCSC Xena database (https://xena.ucsc.edu/), and skeletal muscle samples from the GTEx database (https://www.gtexportal.org/home/) were used as normal controls for the training set, and both data were removing inter-batch differences and normalization. The gene expression data of the validation set for osteosarcoma was downloaded from the GEO database, and the GSE42352 dataset (https://www.ncbi.nlm.nih.gov/geo/query/acc.cgi?acc=GSE42352) was selected from the GEO database as the validation set for this study^10,11^. Subsequently, the probe numbers of the training and validation sets were converted into recognizable gene symbols. here, all statistical analyses and graphing were processed and analyzed using the programming language R x64 (version 4.1.3), using Strewberry Perl (https://strawberryperl.com/, version: v5.32.1) for the conversion of probes into gene symbols and the processing of this paper.

### Differential expression analysis

In this study, we first performed differential expression analysis of genome-wide mRNA expression in the training set using the “limma” package, with cut off values set to |logFC| ≥ 1.5 and adjusted-p < 0.05. We then used the “pheatmap” package to visualize the top 100 differentially expressed genes as heat maps; “dplyr” package, “ggplot2” package and “ggrepel” package to visualize all the differentially expressed genes as a volcano map. To investigate the role of cuproptosis-related genes in OS, 10 cuproptosis genes were extracted from the genome-wide mRNA expression matrix, and each two genes were correlated using the “corrplot” and “circlize” packages and visualized as correlation heat maps.

### Artificial intelligence of randomForest analysis

In order to obtain more accurate early diagnostic biomarkers, a further screening was performed using the randomForest method. Random forest is a supervised learning method in which multiple prediction models are generated simultaneously and the results of the models are aggregated to improve the accuracy^12^. Random forest has many advantages over other AI analysis methods: its ability to handle a large number of input variables, its ability to assess the importance of variables, and its ability to handle mixed data^13,14^. We screened the cuproptosis-associated genes again to obtain the genes for the final diagnostic model.

### Artificial intelligence machine learning-support vector machine recursive feature elimination (SVM-RFE) analysis

Here, we used the SVM-RFE approach to analyze all cuproptosis-related genes in order to obtain the most accurate biomarkers for early diagnosis of OS.

SVM is a class of generalized linear classifiers that perform binary classification of data in a supervised learning manner. The decision boundary is the maximum margin hyperplane solved for the learned samples, i.e., it is the separation hyperplane solved that correctly divides the data set and has the largest geometric separation. SVM-RFE is used to reduce the features and find the optimal number of features, which can remove the confounding factors very efficiently to obtain high accuracy^15^. As an advanced artificial intelligence screening, we analyzed cuproptosis genes using the “e1071” package, the “kernlab” package and the “caret” package of the programming language R to obtain the optimal diagnostic genes.

### Analysis of least absolute shrinkage and selection operator (LASSO) of artificial intelligence

Subsequently, we used LASSO regression analysis for further screening of cuproptosis genes. the LASSO method was able to achieve variable selection by compressing the coefficients of non-significant variables to zero^16^. Therefore, we use this method to obtain the most streamlined and accurate genes for constructing diagnostic models.

### Differentially expressed gene enrichment analysis and protein–protein interaction network (PPI)

In this study, we performed GO enrichment analysis and KEGG pathway enrichment analysis^17–19^ (https://www.kegg.jp/kegg/kegg2.html) for these two genes using GSEA enrichment analysis in order to analyze the role of differentially expressed genes, respectively. The “limma” package, the “org.Hs.eg.db” package, the “clusterProfiler” package and the The “enrichplot” package was used for enrichment analysis of these two genes. Subsequently, to obtain a more accurate PPI network map, we increased the cut off value to 3.2 for another screening, and imported the screened genes into the STRING database (https://cn.string-db.org/) to obtain the relationships among all genes. Subsequently, we identified genes from the PP network that were associated with the diagnostic genes from the screen and constructed their radar maps using the “fmsb” package to visualize their correlation.

### All screening to take the intersection and receiver operating characteristic (ROC) diagnostic curve construction

In this study, we took the intersection of all the screening results and used them to obtain the most accurate and precise genes for the diagnostic model of OS. We used the “VennDiagram” package to take the intersection of the genes screened by the five different methods and visualize it as a Venn diagram. Two genes, Pyruvate Dehydrogenase E1 Subunit Alpha 1 (PDHA1) and Cyclin Dependent Kinase Inhibitor 2A (CDKN2A), were identified in the crossover as genes for the diagnostic model. We used the ROC diagnostic curve approach to examine the diagnostic efficacy of these two genes for the diagnosis of OS. We constructed ROC diagnostic curves for the training and test sets in turn.

### Analysis of tumor immune cell infiltration and correlation between genes and immune cells

In this study, we performed quantitative immune cell analysis of OS samples and normal control samples from the training set using CIBERSORT software^20^ in order to explore in depth the relationship between cuproptosis genes and tumor immune cell infiltration. Tumor immune cell infiltration refers to the transfer of immune cells from the blood to the tumor tissue to begin to exert its effects, and infiltrating immune cells that can be isolated from the tumor tissue. We used the “e1071” package, the “parallel” package and the “preprocessCore” package to analyze immune cell infiltration in OS and control groups. Subsequently, we used the “limma” package, the “reshape2” package, the “ggpubr” package and the “ggExtra” package to correlate 2 genes with immune cells for the construction of the diagnostic model.

### Big data blood test immune cell composition

In this study, we collected samples from a healthy control group and an experimental group diagnosed with osteosarcoma from the First Affiliated Hospital of Guangxi Medical University from January 2012 to January 2022 in order to test the accuracy of immune cells obtained from CIBERSORT software analysis. We counted absolute neutrophil values, neutrophil percentages, absolute lymphocyte values and lymphocyte percentages in a total of 25,665 routine blood data. Among them, there were 1727 OS cases and 23,938 healthy control cases. These four types of data were statistically analyzed and visualized as box plots for this OS group and the healthy control group using a two independent samples *t* test.

### Immunohistochemical specific staining analysis

In the present study, we performed an analysis using immunohistochemistry in order to examine the differences between the two genes used to construct the diagnostic model in OS tissue and in paracancerous tissue. This study was reviewed and approved by the ethics department of the First Affiliated Hospital of Guangxi Medical University for immunohistochemical analysis of tissue samples from anonymous patients, and therefore a waiver of patient informed consent was requested. The participants' informed consent was waived in the name of the review committee of the First Clinical Affiliated Hospital of Guangxi Medical University. The pathological tissue sections used for immunohistochemistry were obtained from OS tissue samples and paracancer tissue samples excised during surgery at the First Clinical Affiliated Hospital of Guangxi Medical University. Antibodies for specific staining of pathological tissues for immunohistochemistry were purchased from Proteintech (PDHA1, https://www.ptgcn.com/products/PDHA1-Antibody-18068-1-AP.htm, item no. 18068-1-AP) and Bioss (CDKN2A, http://www.bioss.com.cn/prolook_03.asp?id=AF08169606000664&pro37=1, item number: bs-0740R). We first dewaxed the completed pathological sections by first immersing the sections in invasive xylene I for 5 min; then 3 times in xylene for 5 min each; then in 95% ethanol for 5 min; in 80% ethanol for 5 min; in 75% ethanol for 5 min; and then rinsing the paraffin in running water for 2 min. The sections were then subjected to steps such as blocking, antigen repair, and blocking bar endogenous peroxidase. We placed the finished stained images under an inverted microscope to observe and collect the images. We found that PDHA1 expression was higher in paraneoplastic tissues than in osteosarcoma. And the expression of CDKN2A was higher in osteosarcoma than in the control group. This is consistent with the results of our analysis.

### Osteosarcoma cell line culture and real-time quantitative-PCR (RT-qPCR)

In this study, all cells used for experimental studies were sourced from the ATCC cell bank (ATCC: The Global Bioresource Center, ATCC). The purchased cells were first resuscitated and subsequently cell passages were performed for all cell lines. Subsequently, PCR primers were designed and synthesized, total RNA was extracted from the experimental and control cells, real-time fluorescence quantitative PCR was performed, reverse transcription was performed, and the raw Ct values obtained by qRT-PCR were substituted into the relative quantification formula (2^−∆∆Ct^) for data analysis.

### Ethical disclosure

This study was approved by the Ethics Review Committee of the First Affiliated Hospital of Guangxi Medical University and was in accordance with the provisions of the Declaration of Helsinki of the World Medical Association.

## Results

### Results of data download and preliminary processing

A total of 88 OS cases were downloaded from the UCSC Xena database and 396 normal skeletal muscle samples were downloaded from the GTEx database, and the data from these two databases were normalized and processed to remove inter-batch differences before synthesizing a dataset as a training set. The GSE42352 downloaded from the GEO database contained a total of 118 samples, including 84 osteosarcoma samples, and a total of 3 osteoblasts were selected as their control group, and the data set of these 87 samples was used as the validation set. We use Perl scripts to transform the probe numbers of the training and validation sets into gene symbols.

### Results of differential expression analysis

**Figure 1:**
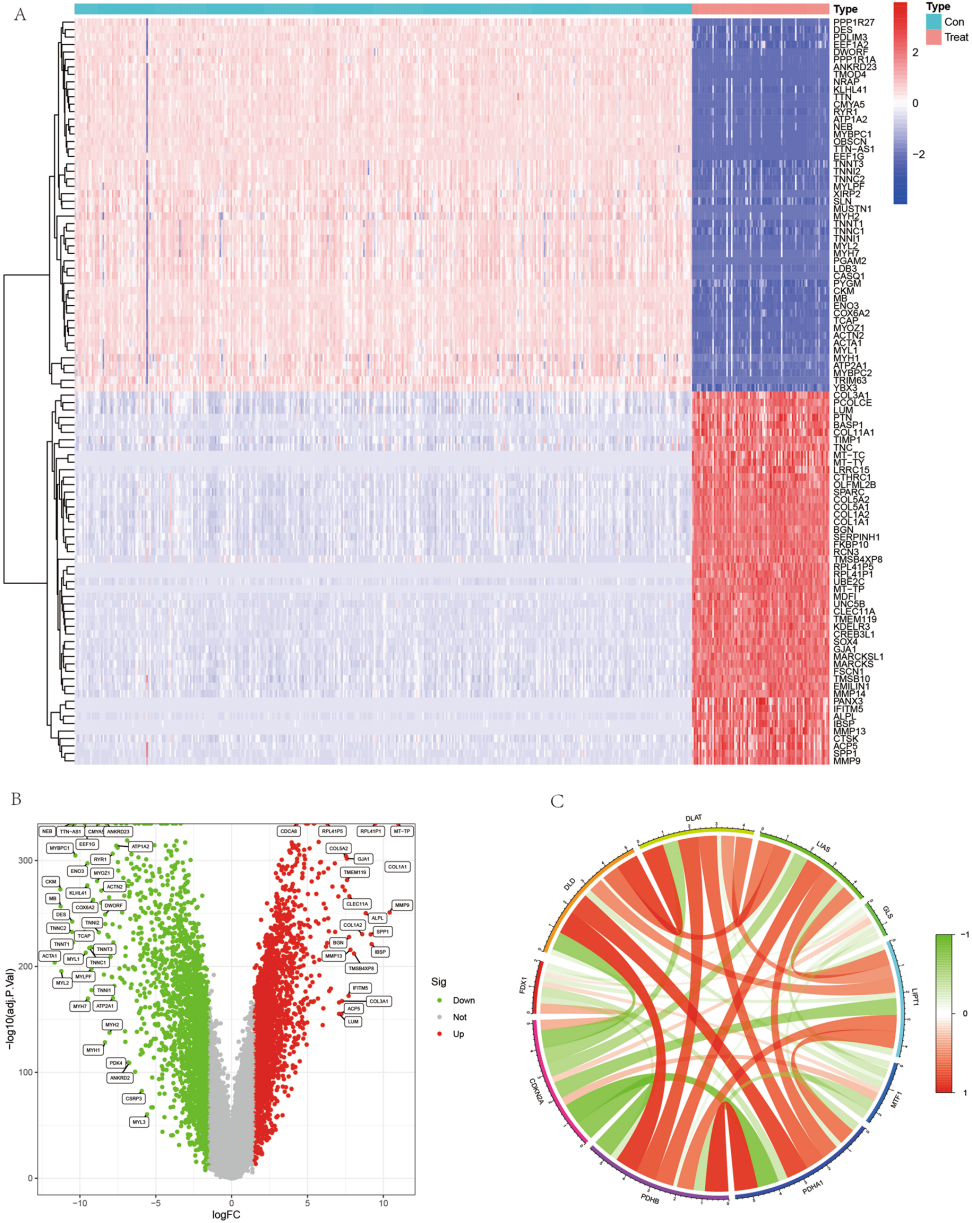
Heat map, volcano plot and correlation heat map of differentially expressed genes. (**A**,**B**) show the heat map and volcano map of differentially expressed genes, respectively, with red indicating highly expressed genes and green indicating lowly expressed genes. (**C**) shows the correlation heat map of cuproptosis-related genes, with the red line indicating synergistic high expression and the green line indicating synergistic low expression.

In this study, we set the cut off value to |logFC| ≥ 1.5, adjusted-p < 0.05, and obtained a total of 4811 differentially expressed genes from the whole genome of 54,751 genes for all genes. We visualized the top 100 differentially expressed genes for the heat map (Fig. 1A) and the volcano map (Fig. 1B). We found that a portion of genes were highly expressed in the OS group and a portion of genes were highly expressed in the control group. Subsequently, we extracted the expression of cuproptosis genes and analyzed the expression relationship between the two two genes, visualized as a correlation heat map (Fig. 1C). From the expression of the correlation heat map we can find that if the line between two genes is red it indicates synergistic high expression and if it is green it is synergistic low expression.

### Results of artificial intelligence stochastic randomForest analysis, SVM-RFE analysis and LASSO regression analysis

**Figure 2:**
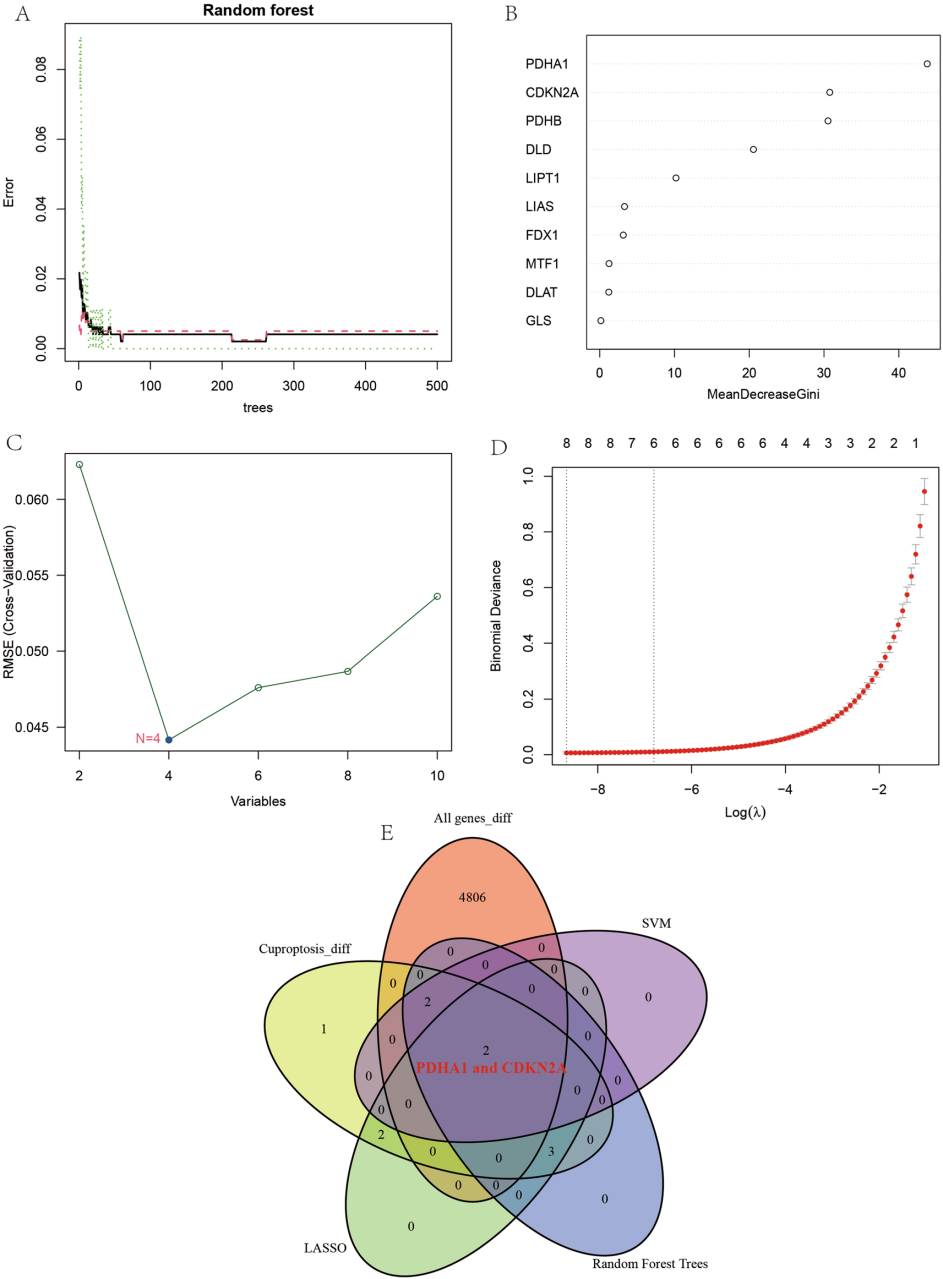
Artificial intelligence screening of osteosarcoma diagnostic genes. (**A**,**B**) show the screening results of the random forest tree. (**C**) shows the results of support vector machine screening. (**D**) shows the results of LASSO regression analysis. (**E**) shows the venn diagram of the intersection of the five screening methods.

In this study, we profiled OS using an artificial intelligence approach in order to obtain more precise diagnostic biomarkers associated with cuproptosis. From the graph of randomForest analysis results (Fig. 2A,B) we can find that PDHA1 and CDKN2A are located in the top 2 positions of the screened genes, i.e. these two genes can be used to get the best efficacy for the diagnosis of OS. From the results of the SVM-RFE analysis (Fig. 2C) we found that the best efficacy was obtained when the number of variable factors was 4, yielding the genes PDHA1, PDHB, CDKN2A and DLD. Subsequently, the genes of the obtained diagnostic model were refined using LASSO regression analysis, and from Fig. 2D we can learn that the best efficacy was obtained when D, we can learn from Fig. 2D that better diagnostic efficacy can be obtained when the number of variables is 6. Finally, we took the intersection of all differential genes, differentially expressed genes associated with cuproptosis, genes obtained by randomForest analysis, genes obtained by SVM-RFE analysis and genes obtained by LASSO regression analysis, and obtained PDHA1 and CDKN2A as the best diagnostic genes associated with cuproptosis in OS (Fig. 2E).

### Results of differentially expressed gene enrichment analysis and construction of PPI

**Figure 3:**
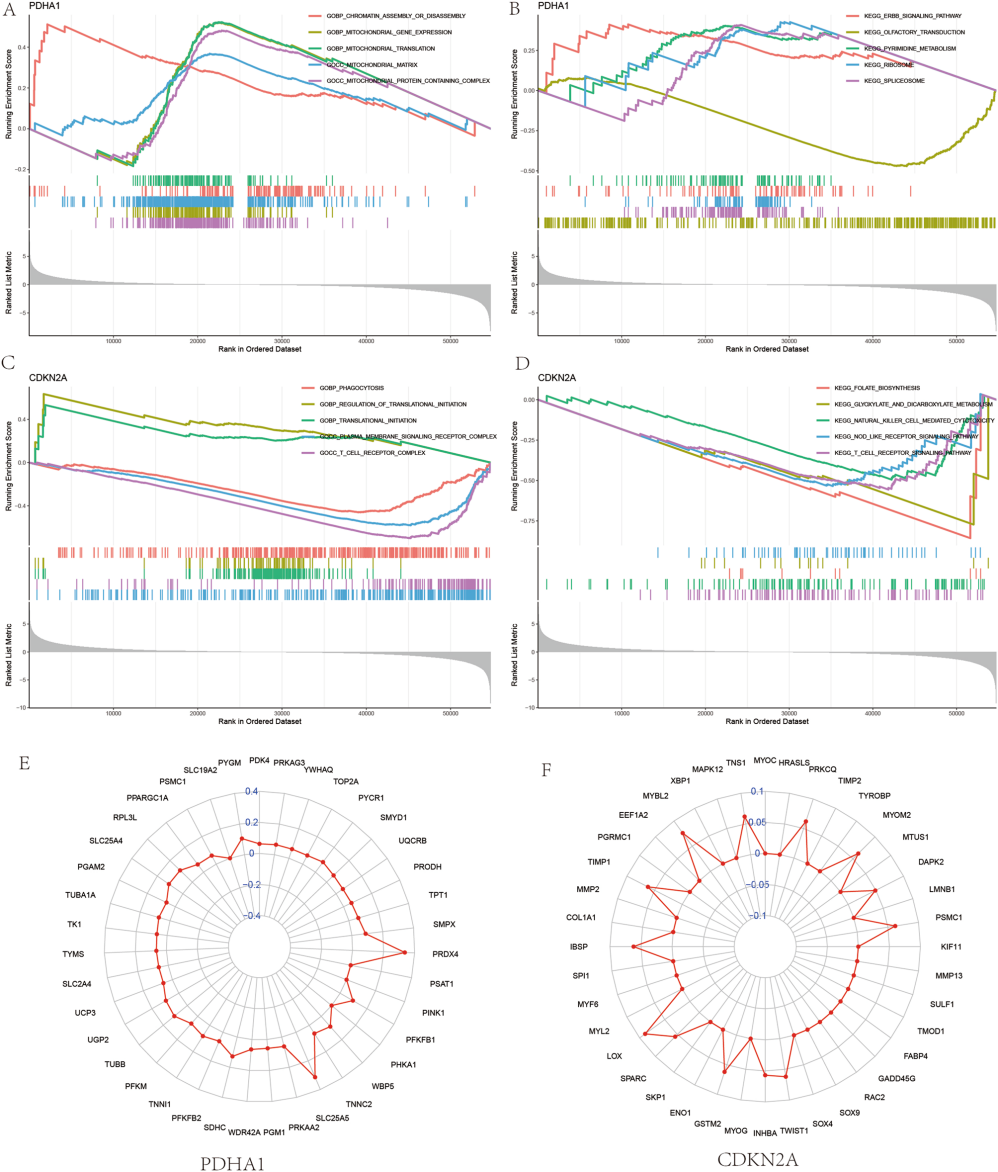
Enrichment analysis of differentially expressed genes. (**A**,**B**) Show the GO enrichment analysis and KEGG pathway enrichment analysis of PDHA1. (**C**,**D**) Show the GO enrichment analysis and KEGG pathway enrichment analysis of CDKN2A. (**E**,**F**) Show the protein–protein interaction network of PDHA1 and CDKN2A. interaction network diagram.

We used the GSEA enrichment analysis method to analyze the 2 genes that construct the OS cuproptosis-related diagnostic model in order to obtain the GO entries and KEGG pathway where the differentially expressed genes are located. We found that the GO entry of PDHA1 (Fig. 3A) was mainly enriched in chromatin assembly or disassembly, mitochondrial gene expression and mitochondrial translation, etc. The KEGG pathway of PDHA1 (Fig. 3B) was mainly enriched in ERBB signaling pathway, pyrimidine metabolism, ribosome and spliceosome. The GO enrichment analysis of CDKN2A was mainly enriched in phagocytosis, Regulation of translation initiation, Translation initiation, Plasma membrane signaling receptor complex and t-cell receptor complexes (Fig. 3C). The KEGG pathway of CDKN2A (Fig. 3D) is mainly enriched in natural killer cell-mediated cytotoxicity, NOD LIKE receptor signaling pathway and T cell receptor signaling pathway. We performed PPI constructs for these two genes and we found that PDHA1 and CDKN2A are closely linked by multiple genes each (Fig. 3E,F).

### Results of ROC diagnostic curve

**Figure 4:**
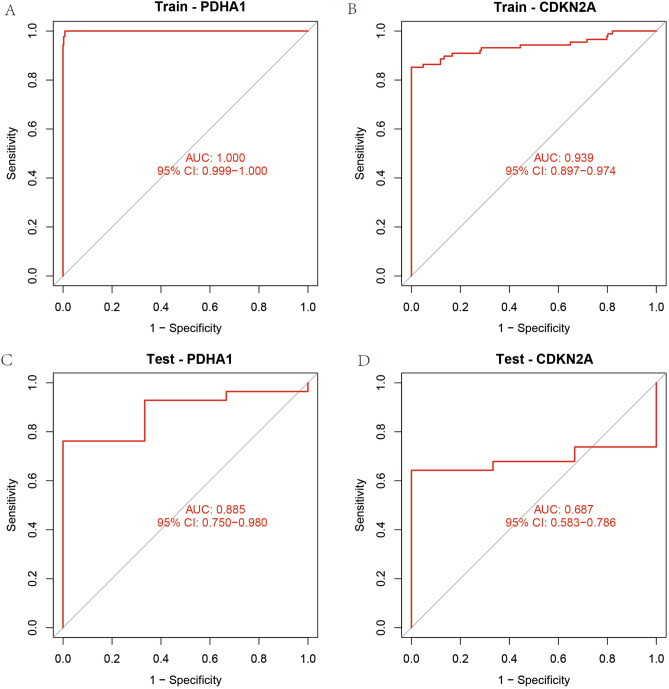
ROC diagnostic curves. (**A**,**B**) Show the ROC diagnostic curves of PDHA1 and CDKN2A in the training set, and it can be found that the results are significantly larger than 0.6. (**C**,**D**) Show the ROC diagnostic curves of the validation set, and the area under the curve is also much larger than 0.6.

In this study, we constructed ROC diagnostic curves in order to test the diagnostic efficacy of the constructed OS cuproptosis-related diagnostic model. From the area under the curve of ROC in the training set (Fig. 4A), we found that the area under the curve of PDHA1 for diagnosing OS reached 100%, while the diagnostic efficacy of PDHA1 in the validation set reached 88.5% (Fig. 4C). On the other hand, CDKN2A was used to diagnose OS with an area under the curve of 93.9% (Fig. 4B), while the diagnostic efficacy of PDHA1 in the validation set reached 68.7% (Fig. 4D). This result illustrates that the OS diagnostic model we constructed is accurate.

### Results of the analysis of tumor immune cell infiltration and correlation between genes and immune cells

**Figure 5:**
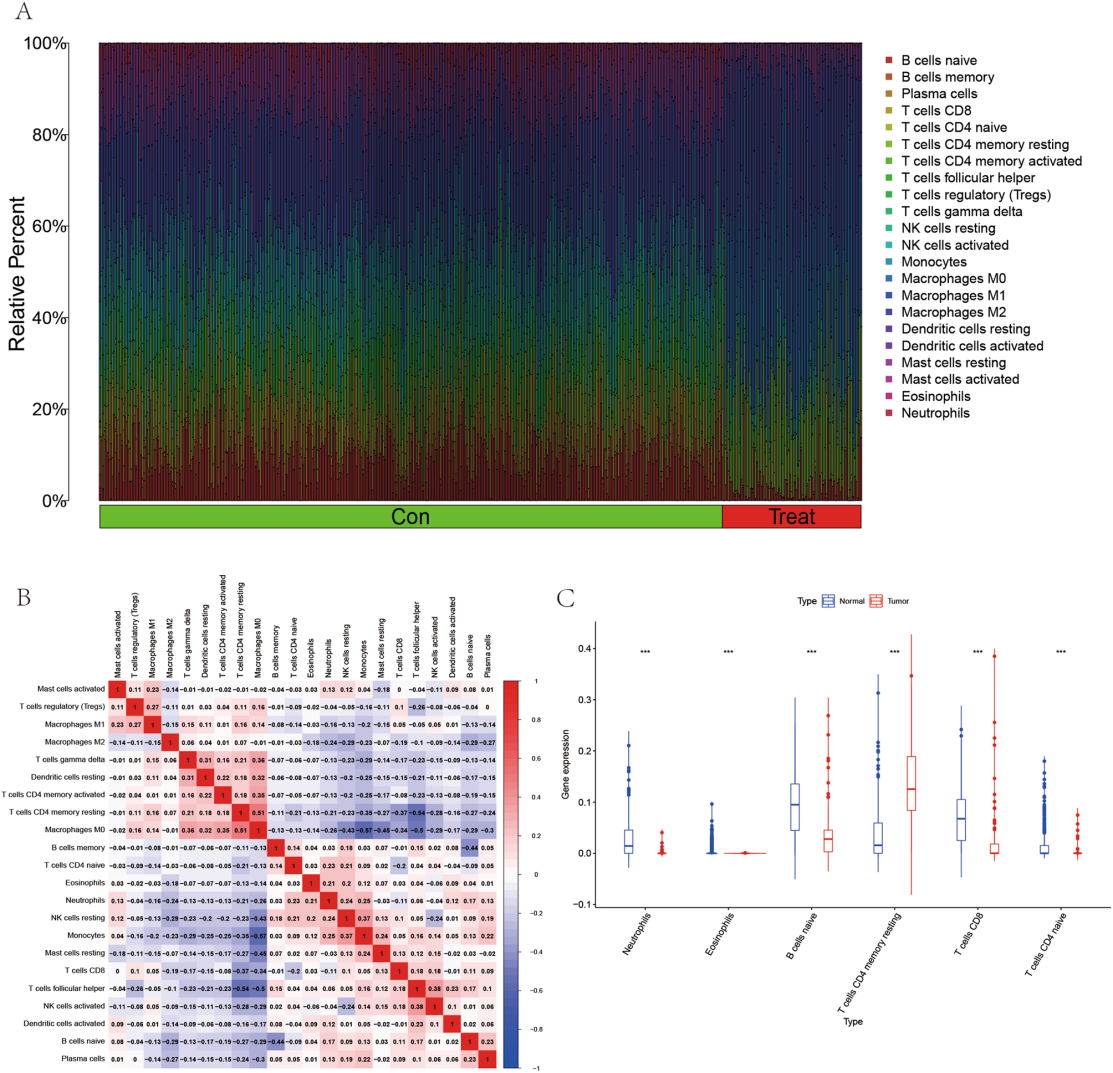
Immune cell infiltration. (**A**) Shows a plot of 22 immune cell compositions for 88 OS and 396 controls. (**B**) Shows a heat map of the correlation between the 22 immune cells, with red indicating synergistic positive correlation and blue indicating synergistic negative correlation. (**C**) Shows a plot of immune cell differences between OS and controls.

In this study, we performed immune cell infiltration and immune cell differential analysis using CIBERSORT software in order to analyze the relationship between OS, a malignant tumor, and immune cell infiltration. From the immune cell composition graph we can find (Fig. 5A) that the immune cell composition of the experimental group (Treat) differs in general from the control group (Con). On the other hand, from the immune cell correlation analysis we can know the correlation between each two immune cells, with red squares indicating a trend of synergistic high expression between these two and blue squares indicating a trend of synergistic ground expression between these two (Fig. 5B). Most importantly, we found from the differential expression analysis of immune cell infiltration that there were multiple immune cell expression differences between OS and normal controls, such as Neutrophils, Eosinophils, B cells naïve, T cells CD4 memory resting, T cells CD8 and T cells CD4 naïve (Fig. 5C).

### Results of gene and immune cell correlation analysis

**Figure 6:**
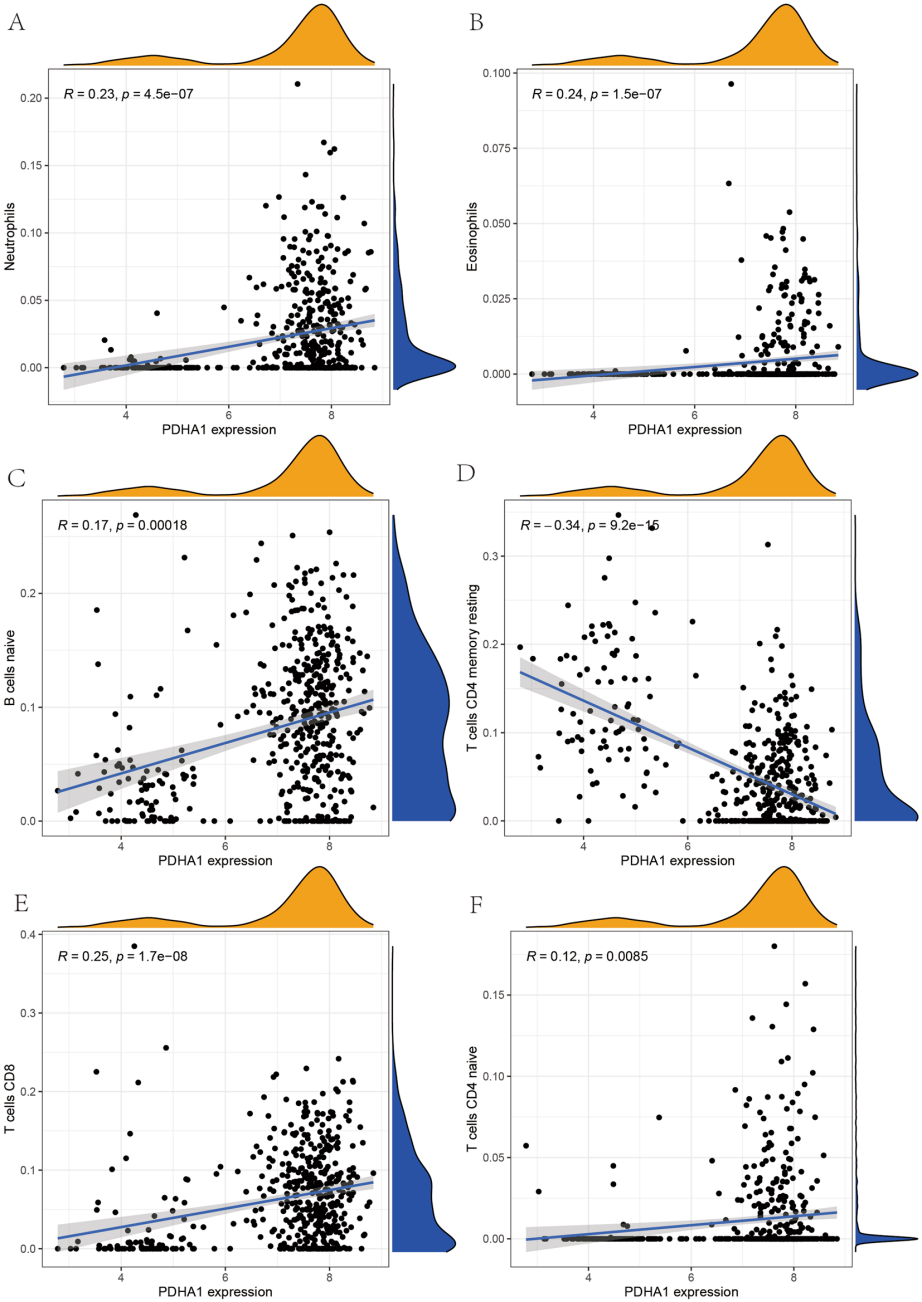
PDHA1 correlation with immune cells. (**A**–**F**) Shows the correlation of PDHA1 gene expression with Neutrophils, Eosinophils, B cells naïve, T cells CD4 memory resting, T cells CD8 and T cells CD4 naïve, respectively.

**Figure 7:**
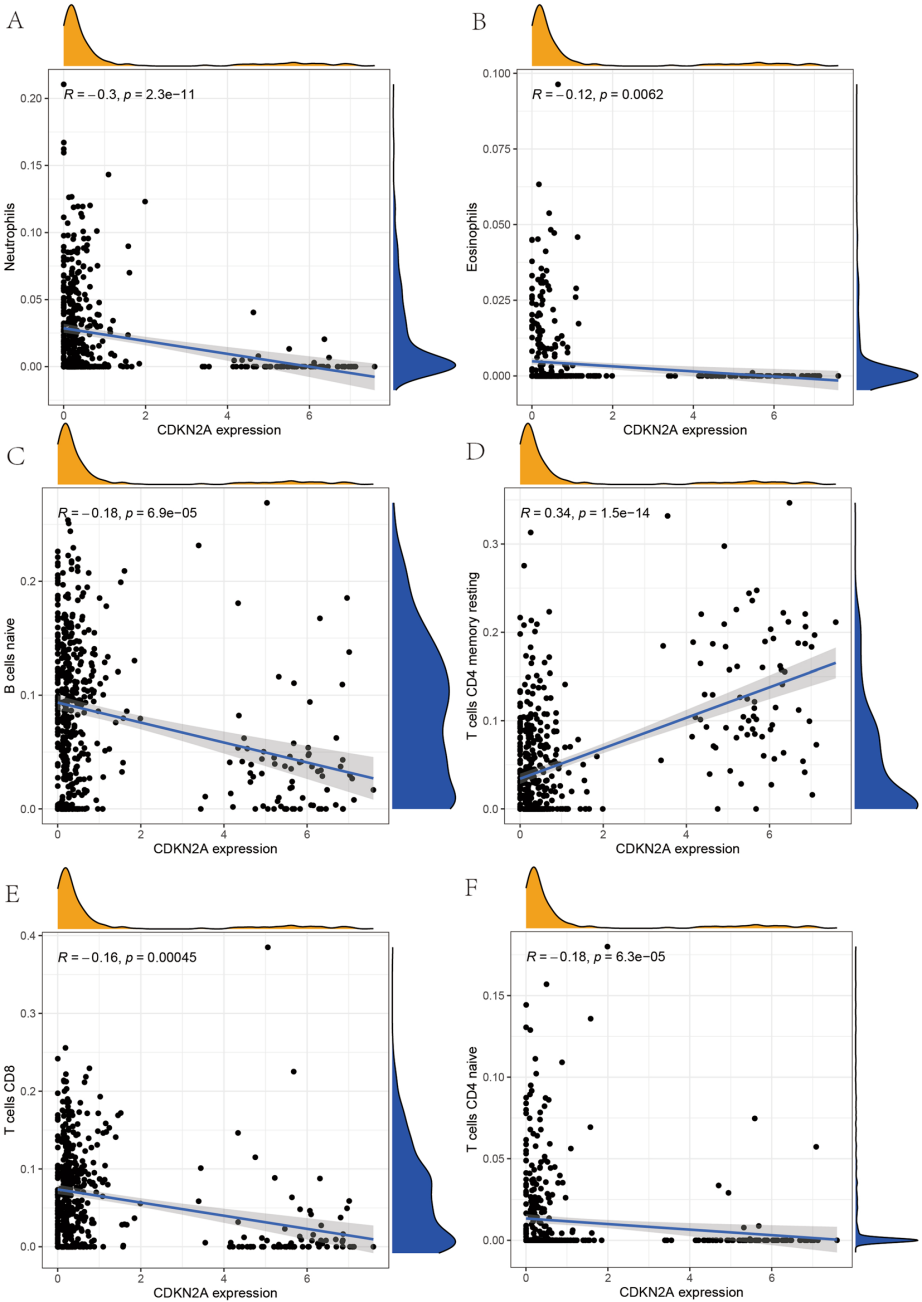
CDKN2A correlation with immune cells. (**A**–**F**) Shows the correlation of CDKN2A gene expression with Neutrophils, Eosinophils, B cells naïve, T cells CD4 memory resting, T cells CD8 and T cells CD4 naïve correlation.

In the present study, we performed a correlation analysis between these two genes and immune cells in order to analyze the relationship between these two genes that construct the OS diagnostic model. We found (Fig. 6A–F) that the gene expression of PDHA1 showed a significant positive correlation with Neutrophils, Eosinophils, B cells naïve, T cells CD8 and T cells CD4 naïve, i.e. the higher the expression of PDHA1, the higher the expression of these immune cells. The gene expression of CDKN2A showed a significant positive correlation with T cells CD4 memory resting and a significant positive correlation with Neutrophils, Eosinophils, B cells naïve, T cells CD8 and T cells CD4 memory resting, cells CD8 and T cells CD4 naïve showed a significant negative correlation (Fig. 7A–F). This result provides a new reference for immunotherapy for the treatment of OS as a malignant tumor.

### Big data to test the results of immune cell differential analysis

**Figure 8:**
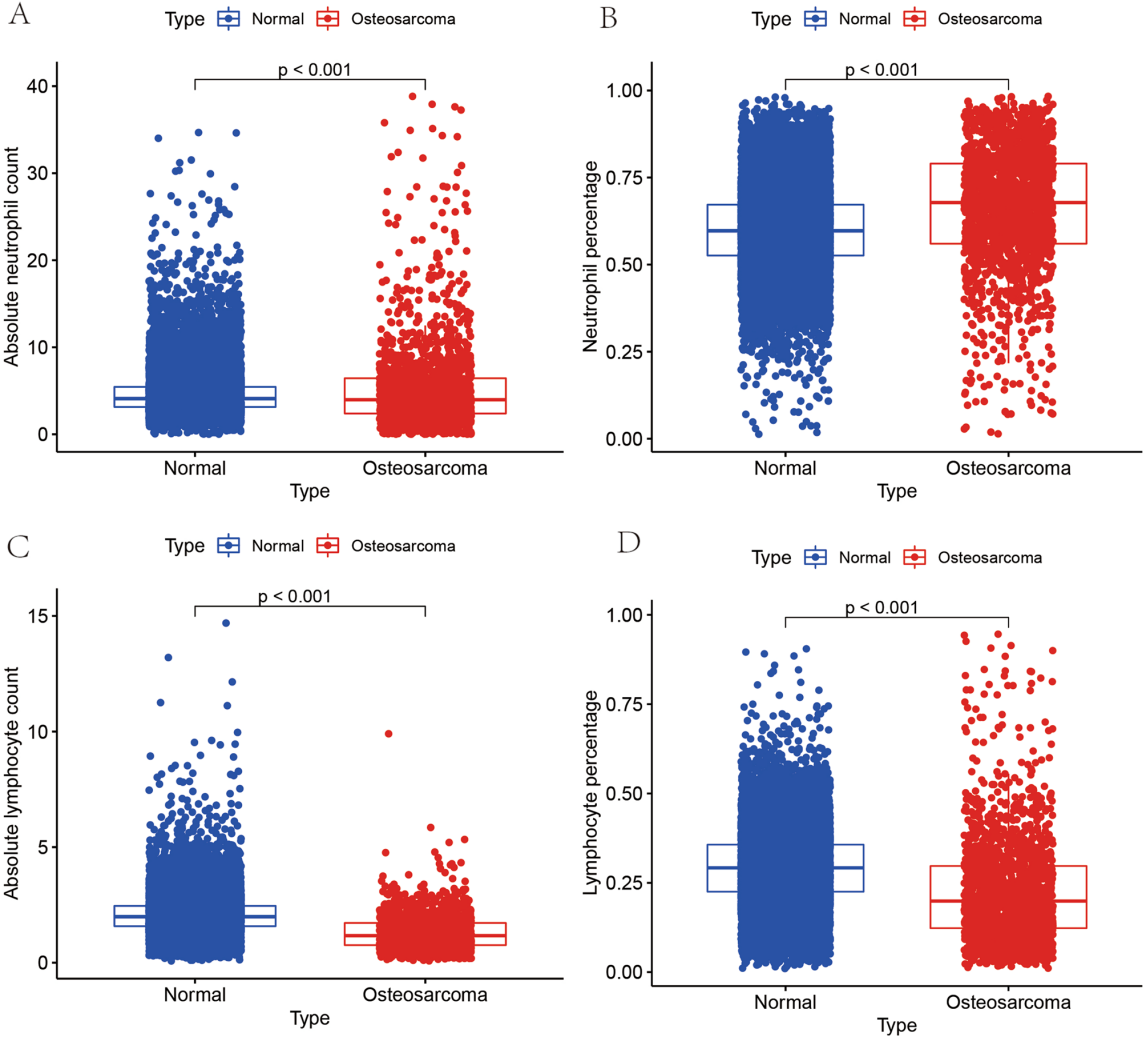
Immunocellular composition of the large data test. (**A**–**D**) Shows the differences in absolute neutrophil values, neutrophil percentages, absolute lymphocyte values, and lymphocyte percentages, respectively, analyzed from routine blood data of a total of 25,665 OS and control cases. The differences are all statistically significant.

In this study, we analyzed absolute neutrophil values, neutrophil percentages, absolute lymphocyte values, and lymphocyte percentages from routine blood data of 25,665 cases (Fig. 8). Our results showed that absolute neutrophil values, neutrophil percentages, absolute lymphocyte values and lymphocyte percentages were significantly different between OS and healthy controls. Moreover, the percentage of neutrophils was significantly higher in cases in the OS group compared to the healthy control group, and the difference was statistically significant. In addition, we also found that the absolute value of lymphocytes and the percentage of lymphocytes were lower in osteosarcoma compared to the healthy control group by the comparison of large data, and the difference was statistically significant. This further illustrates the accuracy of our immune cell infiltration and immune cell differential analysis.

### Results of immunohistochemistry

**Figure 9:**
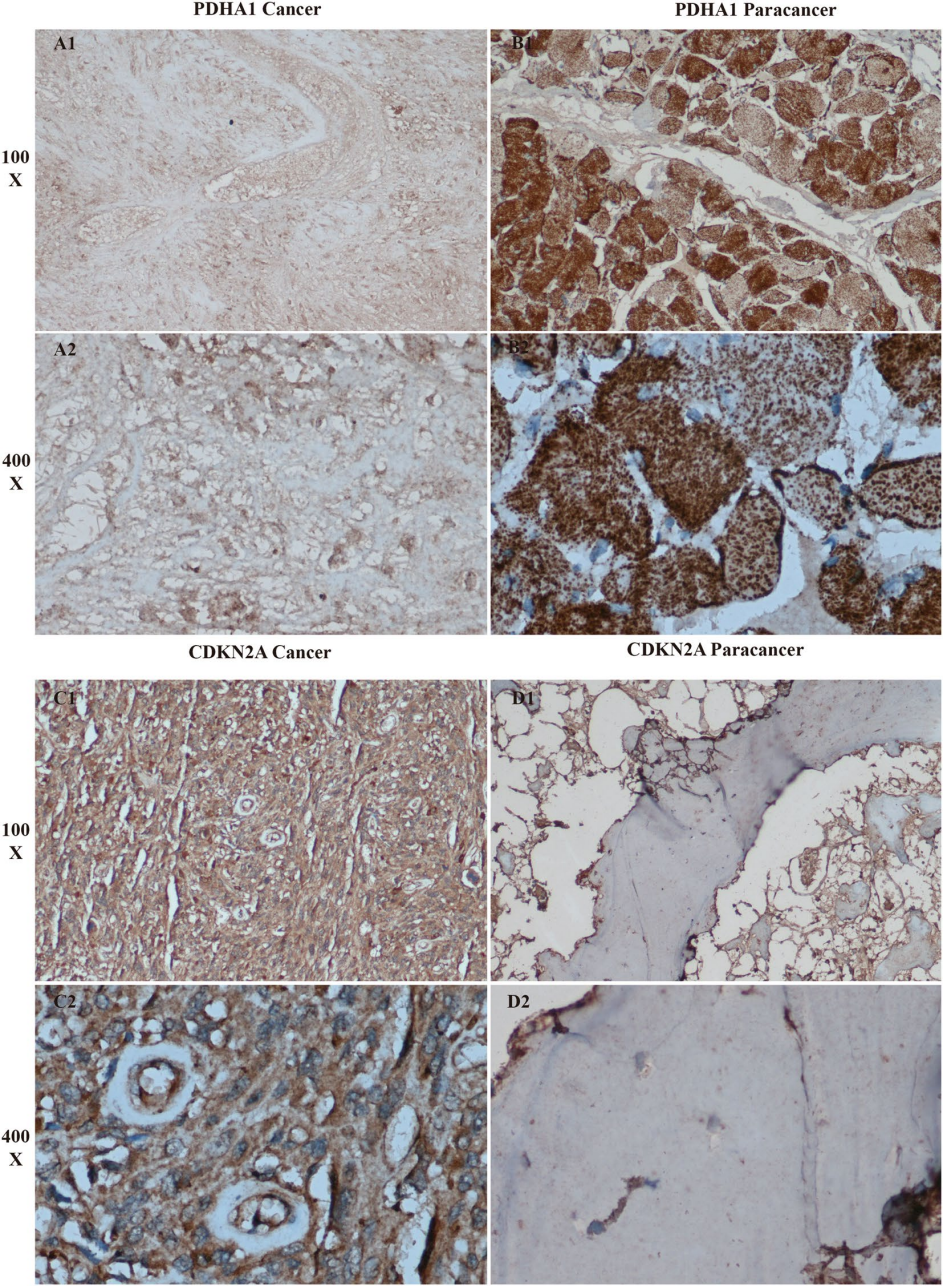
Immunohistochemical specificity analysis. (**A1**–**B2**) Shows the expression of PDHA1 in OS and in paraneoplastic tissues, which shows that the expression of this gene in paraneoplastic tissues is higher than that in OS. (**C1**–**D2**) Shows the expression of CDKN2A in OS and in paraneoplastic tissues, which shows that the expression of this gene in OS is higher than that in in OS than in paraneoplastic tissues.

We performed immunohistochemical specific staining analysis of PDHA1 and CDKN2A, the two genes used to construct the diagnostic model (Fig. 9A1–D2). Their results showed that the expression of PDHA1 was significantly higher in the paraneoplastic tissues than in the OS group. And relative to CDKN2A, its expression was significantly higher in osteosarcoma than in paraneoplastic tissues. This result was consistent with the results of our bioinformatics analysis. The results of bioinformatic difference analysis also showed that CDKN2A was highly expressed in OS, while PDHA1 was highly expressed in paraneoplastic tissues. This further tested the reliability of our analysis.

### Osteosarcoma cell line culture and results of real-time quantitative-PCR (RT-qPCR)

**Figure 10:**
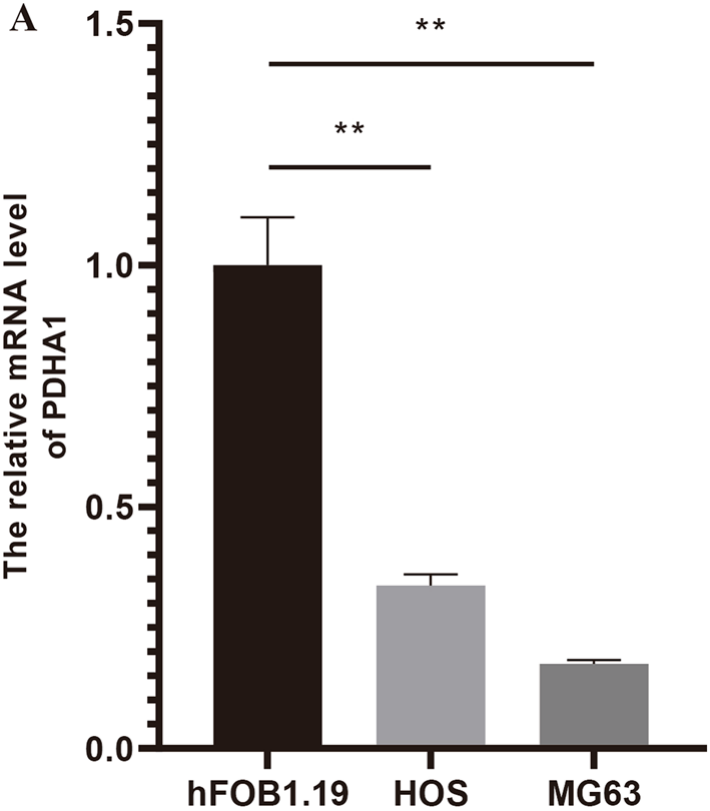
Graph of RT-qPCR results of PDHA1. From the figure, we found that the expression of PDHA1 in control cells was higher than that in OS cell lines.

We obtained the expression of the two genes used to construct the diagnostic model in OS cells and control cells after performing laboratory steps such as resuscitation, passaging, cellular RNA extraction, primer design, and real-time quantitative PCR on cells purchased from the ATCC cell bank. We found from the results that the expression of PDHA1 in control cells hFOB1.19 was significantly lower than that in OS cell lines HOS and MG63, and the difference was statistically significant, which is consistent with our analysis (Fig. 10). This further illustrates the accuracy of our analysis.

## Discussion

In this study, we obtained differentially expressed genes by analyzing their differential expression from the whole genome of OS and controls, and we performed functional enrichment analysis of these genes and found that the KEGG pathway of PDHA1 was mainly enriched in ERBB signaling pathway, pyrimidine metabolism, ribosome and spliceosome. the GO entries of CDKN2A were mainly enriched in phagocytosis, translation initiation and T-cell receptor complex entries. It has been shown that the metabolic reprogramming of beloved signaling promotes cancer development and progression, and notably, one of the causes of cell death is the depletion of pyrimidines^21^. More importantly, macrophages are important mediators of tissue homeostasis, and tumors can distort this propensity to stimulate cell proliferation, metastasis and angiogenesis, in which their phagocytosis also plays an important role^22^. This is consistent with our findings. Our study also showed that two genes containing PDHA1 and CDKN2A were also enriched in the corresponding pathways.

Pyruvate Dehydrogenase E1 Subunit Alpha 1, abbreviated as PDHA1, is a protein-encoded gene, and diseases associated with PDHA1 include pyruvate dehydrogenase E1-alpha deficiency and sudden infant death syndrome^23^. Previous studies have shown that PDHA1 is able to achieve consistent prostate cancer development in human xenograft tumor models by affecting lipid synthesis^24^. In addition, it has also been shown that lysine acetylation of PDHA1 and PDP1 is very common in both epidermal growth factor (EGF)-stimulated cells and various human cancer cells, and that acetylation of K202 is able to inhibit PDP1 by dissociating its fifth PDHA1, both of which have a great role in promoting glycolysis and tumor development in cancer cells^25^. Gonçalves et al. showed that the enzymatic activity of PDHA1 is inhibited by phosphorylation in cells with FH defects, which in turn limits the entry of carbon atoms from glucose into the tricarboxylic acid cycle, and that phosphorylation of PDHA1 is present in tumor cells with FH defects^26^. This is consistent with our findings. Here, we found that two cuproptosis-associated genes, including PDHA1, could be used to diagnose OS with high efficiency and accuracy by exploring novel diagnostic genes for OS by means of artificial intelligence. On the other hand, it has also been shown that reducing pyruvate activity by depleting mitochondrial pyruvate carrier 2 (MPC2) or PDHA1 stimulates and enhances NLRP3 inflammasome activation^27^. This is similar to the results of our study. We performed immune cell infiltration of gene expression in OS and controls by CIBERSROT software and found significant differences in neutrophils and lymphocytes between these two groups, and, we tested this with routine blood data from 25,665 cases. Our study provides a new reference for immunotherapy of OS as a malignancy.

Cyclin Dependent Kinase Inhibitor 2A (CDKN2A) is a protein-encoded gene and diseases associated with CDKN2A include melanoma, cutaneous malignancies 2 and melanoma-pancreatic cancer syndrome^28^. Back in 2016, it has been reported that pancreatic cancer as a malignant tumor, mutation of CDKN2A gene is an important factor for its pancreatic tumorigenesis^29^. It has also been shown that CDKN2A is significantly mutated in cutaneous melanoma in a study by Hayward et al.^30^. It has also been shown that in gliomas, hypermutation and acquired CDKN2A deletion are closely associated with an increase in tumor cells at the time of recurrence of this tumor, and that its changes reflect the active growth state in which the tumor cells are in^31^. More interestingly, this is similar to the results of our study. In the present study, our findings showed significant differential expression of CDKN2A in OS and controls, two cuproptosis-related genes including CDKN2A, which can serve as a diagnostic marker for OS as a malignancy and can guide early diagnosis. On the other hand, the control of cancer by adaptive immunity involves some well-defined clearance and death mechanisms when tumor necrosis factor in combination with interferon-gamma (IFN-γ) drives cancer into senescence by inducing permanent growth arrest in the G1/G0 phase and activation of CDKN2A to drive expression of the gnathostome 40 large T antigen (Tag) expressed under the control of the rat insulin promoter^32^. This is similar to our findings. Our findings suggest that there is a significant dysregulation of neutrophils and lymphocytes in OS and that its may be a key factor with OS.

Here, we screened cuproptosis-related diagnostic genes by three artificial intelligence methods in an attempt to find early biomarkers of OS as a malignant disease and mechanisms related to immune cell infiltration, providing a new basis for early diagnosis and immunotherapy. We screened PDHA1 and CDKN2A as early diagnostic genes for OS using three artificial intelligence methods with high precision and high performance, and both had high diagnostic efficacy. The differential expression of these two genes in OS and control groups was examined by two methods, immunohistochemistry and PCR. The differential expression of this immune cell infiltration was also examined using routine blood data from 25,665 cases.

Of course, our study, like all other studies, has its limitations. First, the inadequacy of sample size, although we used 88 OS and GTEx396 normal controls from UCSC Xena database as the training set and 87 samples from GEO database as the validation set, it is not enough compared to the large sample size. Second, the laboratory validation was insufficient. We only used immunohistochemistry, PCR and clinical large data validation methods for the test.

## Conclusion

PDHA1, CDKN2A, and neutrophils were significantly different in OS and normal controls, and PDHA1, CDKN2A was able to serve as specific biomarkers for OS.

## Data Availability

The datasets used and/or analyzed in the current study are available in the following publicly available datasets. The datasets supporting the conclusions of this article are available in the Ucsc Xena database (http://xena.ucsc.edu/), GTEx Database (https://www.gtexportal.org/home/) and GEO datasets (https://www.ncbi.nlm.nih.gov/gds/).
